# Supplementary material for: Detection and Characterization of Enterovirus B73 from a Child in Brazil
Source: Viruses. 2018 Dec 28;11(1):16. doi: 10.3390/v11010016 (PMC6357135; doi:10.3390/v11010016)
Supplement: Supplementary file 1 [file viruses-11-00016-s001.pdf]

Supplementary material:

Detection and characterization of enterovirus B73 from a child in Brazil

Geovani de Oliveira Ribeiro[1], Adriana Luchs[2], Flávio Augusto de Pádua Milagres [3,4, 5], Shirley Vasconcelos Komninakis [6,7], Danielle Elise Gill[8], Márcia Cristina Alves Brito Sayão Lobato [5,6], Rafael Brustulin[3,4, 5], Rogério Togisaki das Chagas[3,5], Maria de Fátima Neves dos Santos Abrão[3, 5], Cassia Vitória de Deus Alves Soares[3, 5], Steven S. Witkin[8, 9], Fabiola Villanova[1], Xutao Deng[11, 12], Ester Cerdeira Sabino[8,10], Eric Delwart[11, 12], Élcio Leal[1,*,‡] and Antonio Charlys da Costa[8,*,‡]

1 Institute of Biological Sciences, Federal University of Pará, Pará 66075-000, Brazil; fevface@gmail.com (F.V.); geovanibiotec@gmail.com (G.O.R.); elcioleal@gmail.com (É.L.)

2 Enteric Disease Laboratory, Virology Center, Adolfo Lutz Institute, São Paulo 01246-000, Brazil; driluchs@gmail.com

3 Secretary of Health of Tocantins, Tocantins 77453-000, Brazil; flaviomilagres@uft.edu.br (F.A.d.P.M); eumarciaalvesbrito@gmail.com (M.C.A.B.S.L.); eu3rafael@gmail.com (R.B.); chagastogisaki@hotmail.com (R.T.d.C.); fatima_abrao@yahoo.com.br (M.d.F.N.d.S.A.); cassiavitoriaalves@gmail.com (C.V.d.D.A.S.)

4 Institute of Biological Sciences, Federal University of Tocantins, Tocantins 77001-090, Brazil

5 Public Health Laboratory of Tocantins State (LACEN/TO), Tocantins 77016-330, Brazil.

6 Postgraduate Program in Health Science, Faculty of Medicine of ABC, Santo André 09060-870, Brazil; skomninakis@yahoo.com.br (S.V.K.)

7 Retrovirology Laboratory, Federal University of São Paulo, São Paulo 04023-062, Brazil

8 Institute of Tropical Medicine, University of São Paulo, São Paulo 05403-000, Brazil; degill@g.clemson.edu (D.E.G.); sabinoec@gmail.com (E.C.S.); charlysbr@yahoo.com.br (A.C.d.C)

9 Department of Obstetrics and Gynecology, Weill Cornell Medicine, New York, New York, USA;switkin@med.cornell.edu; (S.S.W.)

10 LIM/46, Faculty of Medicine, University of São Paulo, São Paulo 01246-903, Brazil,

11 Blood Systems Research Institute, San Francisco, CA 94143, USA; xdeng@bloodsystems.org (X.D.);eric.delwart@ucsf.edu (E.D)

12 Department Laboratory Medicine, University of California San Francisco, San Francisco, CA 94143, USA

‡These authors jointly supervised this work.

*Correspondence: elcioleal@gmail.com (É.L.); charlysbr@yahoo.com.br (A.C.d.C)

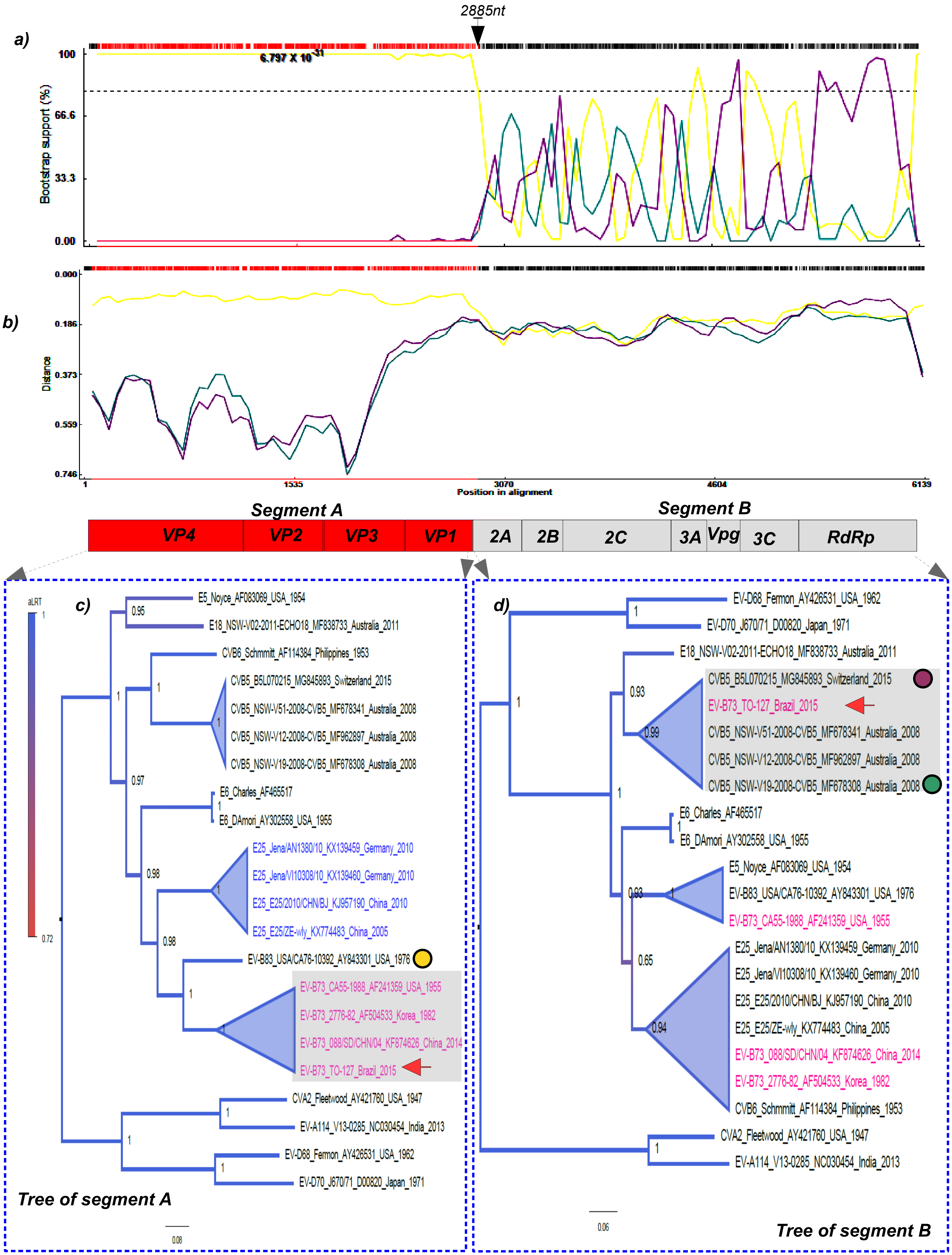

2885nt
a)
100
6.797 X 10^-31
66.6
33.3
0.00
Bootstrap support (%)
b)
0.000
0.186
0.373
0.559
0.746
Distance
1
1535
3070
4604
6139
Position in alignment
Segment A
Segment B
VP4
VP2
VP3
VP1
2A
2B
2C
3A
Vpg
3C
RdRp
c)
aLRT
1
0.72
E5_Noyce_AF083069_USA_1954
E18_NSW-V02-2011-ECHO18_MF838733_Australia_2011
CVB6_Schmitt_AF114384_Philippines_1953
CVB5_B5L070215_MG845893_Switzerland_2015
CVB5_NSW-V51-2008-CVB5_MF678341_Australia_2008
CVB5_NSW-V12-2008-CVB5_MF962897_Australia_2008
CVB5_NSW-V19-2008-CVB5_MF678308_Australia_2008
E6_Charles_AF465517
E6_DAmori_AY302558_USA_1955
E25_Jena/AN1380/10_KX139459_Germany_2010
E25_Jena/VI10308/10_KX139460_Germany_2010
E25_E25/2010/CHN/BJ_KJ957190_China_2010
E25_E25/ZE-wly_KX774483_China_2005
EV-B83_USA/CA76-10392_AY843301_USA_1976
EV-B73_CA55-1988_AF241359_USA_1955
EV-B73_2776-82_AF504533_Korea_1982
EV-B73_088/SD/CHN/04_KF874626_China_2014
EV-B73_TO-127_Brazil_2015
CVA2_Fleetwood_AY421760_USA_1947
EV-A114_V13-0285_NC030454_India_2013
EV-D68_Fermon_AY426531_USA_1962
EV-D70_J670/71_D00820_Japan_1971
Tree of segment A
0.08
d)
EV-D68_Fermon_AY426531_USA_1962
EV-D70_J670/71_D00820_Japan_1971
E18_NSW-V02-2011-ECHO18_MF838733_Australia_2011
CVB5_B5L070215_MG845893_Switzerland_2015
EV-B73_TO-127_Brazil_2015
CVB5_NSW-V51-2008-CVB5_MF678341_Australia_2008
CVB5_NSW-V12-2008-CVB5_MF962897_Australia_2008
CVB5_NSW-V19-2008-CVB5_MF678308_Australia_2008
E6_Charles_AF465517
E6_DAmori_AY302558_USA_1955
E5_Noyce_AF083069_USA_1954
EV-B83_USA/CA76-10392_AY843301_USA_1976
EV-B73_CA55-1988_AF241359_USA_1955
E25_Jena/AN1380/10_KX139459_Germany_2010
E25_Jena/VI10308/10_KX139460_Germany_2010
E25_E25/2010/CHN/BJ_KJ957190_China_2010
E25_E25/ZE-wly_KX774483_China_2005
EV-B73_088/SD/CHN/04_KF874626_China_2014
EV-B73_2776-82_AF504533_Korea_1982
CVB6_Schmitt_AF114384_Philippines_1953
CVA2_Fleetwood_AY421760_USA_1947
EV-A114_V13-0285_NC030454_India_2013
0.06
Tree of segment B

Figure S1. Recombination pattern of enterovirus B73 from Brazil.

The bootscanning method was used to determine the parental genotypes that compose the B73 recombinant strain TO-127 (a). Colored lines represent the probability (given in bootstrap values) of genomic regions to belong to a certain parental genotype. The x-axis represents the sequence length in base pairs (bp). The y-axis represents the statistical support (bootstrap) based on 500 replicates. Each plotted line refers to a certain genotype (see the parental sequence indicated in the trees). The only breakpoint detected in B73 genome is indicated by black arrow and the number 2885 above the arrow is the location of breakpoint in the polyprotein. The upper dashed black and red lines indicate partitions (segment A and segment B) in the alignment that presented conflicting trees. The probability of the breakpoint is also shown below the dash red line ($6.797x10^{-31}$) and it is based on the LARD method that detects recombination breakpoints by scanning an alignment of three sequences (a recombinant and two putative parental sequences) for the point (likelihood ratio test) in the alignment that optimally separates regions of conflicting phylogenetic signal.

Similarity plot method, based on pairwise distances, was also used to determine the proximity of TO-127 to other strains (b). The colored lines show the distances between the B73 TO-127 strain and enterovirus references (see the enterovirus references indicated by colored dots in the trees). The Y-axis indicates the genetic distances, and the x-axis shows the nucleotide positions in the B73 polyprotein. The diagram below the similarity plot is a schematic representation of the polyprotein of enterovirus B. All these analysis were performed using the RDP v4 software)[1] using the evolutionary model Fenselstein, 1984 plus the estimated transition/transversions (ts/tv=1,2). Window sizes of 150 to 350, stepping of 60-150 nt, as well as χ^2 correction with p-values of 0.05 and 0.001 were utilized. Dashed line indicate the cutoff value based on Bonferroni correction test.

Maximum likelihood trees were inferred in alignment partitions indicated by the RDP methods (c, d).

The tree constructed using the partition corresponding to polyprotein region VP4, VP3, VP2 and VP1 (segment A, 2959 bp) indicated that the strain B73-TO-127 (identified by red arrow) belong to a clade formed by B73 strains (indicated by gray area). The tree constructed with the partition corresponding to non-structural genes of enterovirus B (segment B, 3148bp) shows that the strain TO-127 is located at the base of a clade formed by B5 enterovirus strains. Some branches were collapsed to facilitate visualization. Values at nodes indicate the statistical support of trees and were calculated using approximate likelihood ratio test (aLRT). The overall tree support is also show in colors according to the scale in the left side of tree of segment A. Phylogenetic trees were constructed assuming HKY model plus gamma correction, FastTree [2] software was used to these inferences. Edition and visualization of tree were made using FigTree version 1.4 software (available at:http://figtree.googlecode.com).

Methodology

Detection of recombination

To determine the extent of recombination among sequences, we used software RDP v.4 (Martin et al., 2015), which utilizes a collection of methods. Below is a brief description of these methods; an excellent and detailed explanation of each method implemented in the RDP program can be found in the user's manual (http://darwin.uvigo.es/rdp/rdp.html). To estimate recombination rates the INTERVAL program (within the RDP program) was used; it estimates variations in recombination rates along an alignment using a penalized approximate likelihood approach within a Bayesian reversible-jump Markov chain Monte Carlo (RJMCMC) approach. The initial estimate of the alignment-wide population scaled recombination rate (rho as a starting point) was the default and then we evaluated distinct initial values (0–30) to see if it could affect the results. 3Seq performs an exact nonparametric test to detect recombination in triplets of sequences. Maximum $\chi 2$ (MaxChi) is a method implemented by Maynard-Smith, and it uses variable/invariable sites to detect recombination in pairs of sequences. This method generates random sequence pairs; the significance level is evaluated by the proportion of simulated sequence pairs with maximum $\chi 2$ values higher than the real data. The maximum match $\chi 2$ (Chimaera) is a modification of Smith's method. It uses variable sites to calculate the maximum $\chi 2$ match statistics. Geneconv detects gene conversions (recombination) by evaluating conserved substitutions in fragments between two sequences. Although evolutionary methods are not explicitly implemented in Geneconv, it is robust and has low levels of false positive detection of recombination, including those events due to rate heterogeneity and natural selection. Bootscanning is a sliding window method that was developed to identify the parental origins of sequence fragments (windows) within known or putative recombinant sequences. Lard is similar to MaxChi, and the method scans an alignment of three sequences (a recombinant and two parental sequences) for the point in the alignment that optimally separates regions of conflicting phylogenetic signals; p-values are also estimated to the breakpoint. Initially, we used default parameters; we later optimized the parameters to avoid detection of false positive recombination. In addition, window sizes of 50 to 350, stepping of 50-100nt, as well as Bonferroni correction with p-values of 0.05 and 0.001 were utilized.

References:

1) Martin, D. P., Murrell, B., Golden, M., Khoosal, A. & Muhire, B. RDP4: Detection and analysis of recombination patterns in virus genomes. Virus Evol 1, vev003, doi:10.1093/ve/vev003 (2015).

2) Price, M. N., Dehal, P. S. & Arkin, A. P. FastTree 2--approximately maximum-likelihood trees for large alignments. PLoS One 5, e9490, doi:10.1371/journal.pone.0009490 (2010)
